# Efficacy of Various Tuberculosis Treatment Regimens at a Tertiary Health Care Center in South India: A Retrospective Study

**DOI:** 10.7759/cureus.64496

**Published:** 2024-07-13

**Authors:** Purnima Mariah Benedict Raj, Gurupavan Kumar Ganta, Carmelin Durai Singh, Raman Muthusamy

**Affiliations:** 1 Centre for Global Health Research, Saveetha Medical College and Hospital, Saveetha Institute of Medical and Technical Sciences, Saveetha University, Chennai, IND; 2 Department of Biochemistry, Saveetha Medical College and Hospital, Saveetha Institute of Medical and Technical Sciences, Saveetha University, Chennai, IND

**Keywords:** retrospective study, drug resistance, drug efficacy, treatment regimens, mtb (mycobacterium tuberculosis)

## Abstract

Introduction

Tuberculosis (TB) infection continues to be a major chronic infection causing significant morbidity and mortality, despite being a preventable and treatable infectious disease. The emergence and rapid spread of drug-resistant strains of *Mycobacterium tuberculosis* (MTB), the causative bacteria, present a formidable challenge to global TB control efforts. This study aimed to estimate the efficacy of TB treatment regimens and their successful outcomes in a retrospective analysis carried out in a tertiary health care hospital.

Materials and methods

A retrospective analysis was carried out on the patients diagnosed with TB and treated with different treatment regimens at Saveetha Medical College and Hospital (SMCH), Chennai, India, between November 2022 and July 2023. Data were collected on demographics, clinical characteristics, treatment regimens, and treatment outcomes of the above patients.

Results

A total of 234 patients were included in the study. The patients were divided according to sex, age, and resistant characteristics; the statistical significance of the collected population was determined. Treatment regimens were followed as either a six-month regimen or nine-month regimen.

Conclusion

This study provides insights into the comparative efficacy of two TB treatment regimens. The findings highlight the importance of proper analysis of the resistance status of the drug and the initiation of medication over an appropriate period of time.

## Introduction

Tuberculosis (TB) is a serious contagious disease caused by airborne gram-positive bacilli called *Mycobacterium tuberculosis* (MTB). The bacteria spread from person to person when someone with active TB in their lungs or throat coughs or sneezes. TB primarily affects the lungs, but it has the capacity to spread to other organs like the spine, brain, and kidneys. Therefore, it is crucial to be aware of the symptoms and seek medical attention promptly to prevent transmission and ensure a successful recovery [1]. Though the development of effective antibiotics marked a significant step forward in the fight against TB, the disease continues to pose a serious threat to public health, especially in low- or middle-income countries. The World Health Organization (WHO) report highlights that in 2023, it was estimated that about 192 countries and areas with more than 99% of the world’s population, including TB-burden countries, reported having TB cases [2]. According to the “India TB 2023” report [3], the country witnessed a significant increase in reported cases of TB, with 24.2 lakh cases indicating a 13% increase compared to the previous year. Therefore, approximately 172 individuals per 100,000 people in the population are diagnosed with TB. The rise in case reports is attributed to multidrug-resistant tuberculosis (MDR-TB), which poses a major challenge.

Research suggests that the escalation of MDR-TB is due to mutations in resistant strains of MTB, which make the treatment ineffective, as the bacteria become less susceptible to drugs [4]. This occurs through various mechanisms, namely, changes in the target of drugs, efflux pump activation on the bacterial surface, obstruction in drug activation, and difficulty in the production of drug-deactivating enzymes [5]. While the global burden remains unclear, accurate assessments of MDR-TB prevalence are essential for initiating an appropriate treatment regimen. There are many hidden costs for patients in India, who incur several "out-of-pocket" expenses, including physician charges, the purchase of non-TB drugs, and convenience charges during the medication period [6]. Nevertheless, nutritional supplements and additional foods remain consistent for all patients undergoing anti-TB treatment. Although the Indian government continues to list financial risk protection as one of its objectives for achieving universal health coverage, estimating such catastrophic costs remains challenging [7].

Assessment by both national and international health organizations is essential for developing effective strategies and improving MDR-TB management within existing TB control programs. Notably, in limited-resource settings, treatment for MDR-TB has shown promising results, which gives hope for tackling this growing public health threat [8]. Hence, evaluating the treatment outcomes of specific regions is crucial for addressing TB control problems in those areas. With this concept, the study focused on the Tiruvallur district, which adjoins the greater Chennai Corporation limits in the South Indian state of Tamil Nadu. Even though effective treatment regimens exist, achieving optimal outcomes relies on various factors. Limited data exist on treatment outcomes and associated risk factors within this specific locality. Therefore, this study aims to evaluate the TB treatment regimens of patients receiving care in tertiary healthcare settings within the Tiruvallur district of Tamil Nadu. Additionally, the study seeks to identify factors influencing and contributing to improved TB control strategies in this region.

## Materials and methods

Study design and participants

To assess the effectiveness of different TB treatment regimens, a retrospective cohort analysis study was conducted for 234 patients diagnosed with pulmonary and extrapulmonary TB infection. The study spanned two regimens: the first, a six-month regimen comprising patients initiating treatment between November 2022 and April 2023; and the second, a nine-month regimen, with patients initiating their treatment between November 2022 and July 2023. The necessary approval was obtained from the Institutional Review Board for carrying out this study program (IRB No. 112101147/SMC/SIMATS).

Sample collection

The study included patients undergoing treatment for confirmed cases of TB during the period from November 2022 to July 2023 at Saveetha Medical College and Hospital (SMCH), Chennai, India. These patients were declared positive for TB infection with isolation of MTB from different regions of the respiratory system, such as the laryngeal, pharyngeal, and lungs. The study also included patients with confirmed cases of TB of non-respiratory origin, such as the intestines, spinal cord, and lymph nodes.

Methodology

The collected samples of pulmonary TB include nasopharyngeal swabs, oropharyngeal swabs from the upper respiratory tract, and expectorated sputum samples collected by inducing a deep cough. Extrapulmonary TB samples included cerebral spinal fluid obtained by lumbar puncture, pleural fluid collected by thoracentesis, pus samples from wound exudates, and blood samples obtained by vein puncture. The collected samples were subjected to comprehensive analysis at SMCH. Mycobacterial culture was subjected to a phenotypic drug susceptibility test (DST) on both solid and liquid media (Löwenstein-Jensen (LJ)), which contained the critical concentration of anti-TB drugs at 37°C. Susceptibility and resistance were analyzed based on the growth or inhibition of the mycobacterial culture. The anti-TB drugs included the first-line drugs rifampicin and isoniazid, and some second-line drugs such as kanamycin, amikacin, ofloxacin, and levofloxacin [9]. After confirmation of the mycobacterial culture as MDR, genotypic DST was performed using the nucleic acid amplification test (NAAT) GeneXpert [10]. Based on the resistant characteristics, the treatment regimens followed were six-month and nine-month regimens.

Statistical analysis

The study utilized IBM SPSS Statistics for Windows, Version 23 (Released 2015; IBM Corp., Armonk, NY, USA) to analyze clinically relevant data. Continuous variables were summarized as mean ± standard deviation (mean ± SD) and compared between groups using the independent sample t-test. For categorical variables, the two-way Chi-squared (χ²) test was employed. Statistical significance was established at a p-value ≤ 0.05.

Treatment regimens

Six-Month Treatment Regimen

Based on the findings of the TB-PRACTECAL [9,11] and ZeNix trials [12,13], the six-month treatment regimen (BPaLM: bedaquiline, pretomanid, linezolid, and moxifloxacin) was recommended for patients with MDR-TB or extensively drug-resistant tuberculosis (XDR-TB). Notably, BPaLM offers a potentially shorter treatment duration of 26 weeks (six months). If fluoroquinolone resistance is detected after treatment initiation, moxifloxacin should be discontinued, and the treatment plan should be continued as BPaL (without moxifloxacin) [14]. This new regimen offers a shorter treatment duration (six months) compared to existing options, potentially improving patient outcomes and adherence (Table 1) [15].

**Table 1:** Six-month treatment regimen (MDR/RR-TB BPaLM/BPaL/BPaLC) For patients with MDR-TB or RR-TB initiated with the BPaL regimen the treatment can be extended to a total of nine months (39 weeks) if their sputum cultures remain positive between four and six months MDR/RR-TB: Multi-drug resistant tuberculosis/Rifampicin-resistant tuberculosis; BpaLM: Bedaquiline, pretomanid, linezolid (600 mg) and moxifloxacin; BPaL: Bedaquiline, pretomanid, linezolid; BPaLC: Bedaquiline, pretomanid, linezolid and clofazimine

SI. No.	Six-month treatment regimen (MDR/RR-TB BPaLM/BPaL/BPaLC)	Antibiotics	Duration and direction of use
1	Sensitive to fluoroquinolones	BPaLM	600 mg once daily for 26 weeks
2	Resistant to fluoroquinolones	BPaL	Discontinuation of moxifloxacin and continued as BPaL
3	Resistant to bedaquiline, pretomanid, linezolid	Longer individualized regimen	Discontinuation of BPaLM/BPaL
4	TB-PRACTECAL trail	BPaL, BPaLM, and BPaLC	Six-month regimen (26 weeks)
5	ZeNix trail	Linezolid	600 mg once daily for 26 weeks

Nine-Month Treatment Regimen

For patients confirmed with MDR-TB or XDR-TB who are not eligible for the BPaLM nine-month regimen, an alternative treatment was opted for. Two phases of treatment were carried out during the nine months of DR-TB treatment. During the first intense phase, which lasted four to six months, bedaquiline was used in combination with high-dose pyrazinamide and clofazimine, in addition to several types of antibiotics, including fluoroquinolones, ethionamide, ethambutol, and isoniazid. This was followed by a five-month continuation phase focusing on fluoroquinolones, clofazimine, ethambutol, and pyrazinamide. Notably, a nine-month regimen is preferred for patients with DR-TB who have not received second-line treatments, including bedaquiline (Table 2) [14].

**Table 2:** Nine-month treatment regimen (MDR/RR-TB BPaLM/BPaL/BPaLC) MDR/RR-TB: Multi-drug resistant tuberculosis/Rifampicin-resistant tuberculosis; BpaLM: Bedaquiline, pretomanid, linezolid (600 mg) and moxifloxacin; BPaL: Bedaquiline, pretomanid, linezolid; BPaLC: Bedaquiline, pretomanid, linezolid and clofazimine

SI. No.	Nine-month treatment regimen (MDR/RR-TB BPaLM/BPaL/BPaLC)	Antibiotics	Duration and direction of use
1	Intensive phase: sensitive to fluoroquinolones	Bedaquiline in combination with levofloxacin/moxifloxacin, ethionamide/linezolid, ethambutol, isoniazid, pyrazinamide and clofazimine	4-6 months dosage of linezolid 600 mg once daily for 39 weeks
2	Continuation phase: sensitive to fluoroquinolones	Fluoroquinolones, clofazimine, ethambutol, pyrazinamide	5 months (39 weeks)

Inclusion criteria

In the present study, patients were selected based on the following inclusion criteria: patients aged 16-55 years, patients confirmed as positive for TB by phenotypic testing, and patients confirmed with MDR and/or XDR-TB infection. Prior history of treatment regimens, such as rifampicin, isoniazid, fluoroquinolone, and a short-course treatment regimen of six months, were included.

Exclusion criteria

Patients with severe pre-existing conditions, such as organ failure, uncontrolled Human Immunodeficiency Virus/Acquired Immunodeficiency Syndrome (HIV/AIDS), or severe active malignancy, may be excluded due to the potential increased risk of complications. Pregnant or breast-feeding women are excluded due to potential teratogenicity or drug transfer to the fetus or infant. Additionally, patients with severe, unbalanced psychological conditions, such as non-cooperation with the treatment, can be excluded from the treatment regimen.

## Results

The treatment outcomes of cases from both the six-month and nine-month treatment regimens are summarized and presented in Table 3. The treatment given to each individual was categorized by sex, age in years, cases transferred to the TB ward, MDR-TB, pre-DR-TB, and fluoroquinolone-resistant and sensitive cases. Out of 234 TB patients registered at SMCH between November 2022 and July 2023, 207 samples were found to be positive, and 27 samples were negative.

**Table 3:** Respiratory and non-respiratory samples collected between November 2022 and July 2023 n%: Samples collected in percent

SI. No.	Types of samples collected	Number of samples collected (n%)	Respiratory/non-respiratory	Positive (n%)	Negative (n%)
1	Nasopharyngeal swabs	47 (20%)	Respiratory	44 (21.2%)	3 (11.11%)
2	Oropharyngeal swabs	39 (16.6%)	Respiratory	32 (15.4%)	7 (25.9%)
3	Sputum	36 (15.3%)	Respiratory	31 (14.9%)	5 (18.5%)
4	Spinal fluid	24 (10.2%)	Non-respiratory	22 (10.6%)	2 (7.4%)
5	Pleural fluid	21 (8.9%)	Non-respiratory	19 (9.17%)	2 (7.4%)
6	Blood	24 (10.2%)	Non-respiratory	20 (9.6%)	4 (14.8%)
7	Pus	43 (18.37%)	Non-respiratory	39 (18.8%)	4 (14.8%)
8	Total	234	-	207	27

The number of patients was categorized as those undergoing ongoing treatment and those who recovered. Among the 207 positive samples, 115 patients received a six-month treatment regimen (Table 4), and 92 patients received a nine-month treatment regimen (Table 5).

**Table 4:** Patients underwent a six-month treatment regimen between November 2022 and April 2023 The data has been presented as n%, mean ± SD, and a p-value ≤ 0.05 is considered significant Total No. of samples = 115 TB: Tuberculosis; MDR-TB: Multi-drug resistant tuberculosis; XDR-TB: Extensively drug-resistant tuberculosis; n%: Number of patients in percent

Category		Ongoing treatment (n%)	Patients recovered (n%)	Total (n%)	p-value
Sex	Male	24 (20.8%)	48 (41.7%)	72 (62.6%)	0.3602
	Female	18 (15.6%)	25 (21.7%)	43 (37.3%)	
Age (years)	16-25	7 (6%)	14 (12.1%)	21 (18.2%)	0.0411
	26-35	13 (11.3%)	13 (11.3%)	26 (22.6%)	
	36-45	5 (4.3%)	28 (24.3%)	33 (28.6%)	
	46-55	12 (10.4%)	23 (20%)	35 (30.4%)	
Patient category	Cases transferred to TB ward	7 (6%)	6 (5.2%)	13 (11.3%)	0.9292
	MDR-TB	16 (13.9%)	16 (13.9%)	32 (27.8%)	
	Pre-XDR-TB	11 (9.5%)	13 (11.3%)	24 (20.8%)	
	Fluoroquinolone resistant	16 (13.9%)	13 (11.3%)	29 (25.2%)	
	Fluoroquinolone sensitive	10 (8.6%)	7 (6%)	17 (14.7%)	

**Table 5:** Patients underwent a nine-month treatment regimen between November 2022 and July 2023 The data has been presented as n%, mean ± SD, and a p-value ≤ 0.05 is considered significant Total No. of samples = 92 TB: Tuberculosis; MDR-TB: Multi-drug resistant tuberculosis; XDR-TB: Extensively drug-resistant tuberculosis; n%: Number of patients in percent

Category		Ongoing treatment (n%)	Patients recovered (n%)	Total (n%)	p-value
Sex	Male	21 (22.8%)	39 (42.3%)	60 (65.2%)	0.0255
	Female	19 (20.6%)	13 (14.1%)	32 (34.7%)	
Age (years)	16-25	3 (3.2%)	8 (8.6%)	11 (11.9%)	0.1682
	26-35	7 (7.6%)	8 (8.6%)	15 (16.3%)	
	36-45	8 (8.6%)	26 (28.2%)	34 (36.9%)	
	46-55	15 (16.3%)	17 (18.4%)	32 (34.7%)	
Patient category	Cases transferred to TB ward	5 (5.4%)	7 (7.6%)	12 (13%)	0.6952
	MDR-TB	14 (15.2%)	10 (10.8%)	24 (26%)	
	Pre-XDR-TB	13 (14.1%)	14 (15.2%)	27 (29.3%)	
	Fluoroquinolone resistant	8 (8.6%)	4 (4.3%)	12 (13%)	
	Fluoroquinolone sensitive	9 (9.7%)	7 (7.6%)	16 (17.3%)	

Out of both males and females, 45 (21.73%) males were undergoing ongoing treatment, and 87 (42%) had completed and recovered from TB. Among female patients, 37 (17.8%) were undergoing ongoing treatment, and 38 (18.3%) had completed and recovered from TB. In total, 132 (63.7%) male and 65 (31.4%) female patients were presented at the hospital (Table 6).

**Table 6:** Total number of patients who received the treatment regimen between November 2022 and July 2023 The data has been presented as n%, mean ± SD, and a p-value ≤ 0.05 is considered significant Total No. of samples = 207 TB: Tuberculosis; MDR-TB: Multi-drug resistant tuberculosis; XDR-TB: Extensively drug-resistant tuberculosis; n%: Number of patients in percent

Category		Ongoing treatment (n%)	Patients recovered (n%)	Total (n%)	p-value
Sex	Male	45 (21.73%)	87 (42%)	132 (63.7%)	0.0316
	Female	37 (17.8%)	38 (18.3%)	65 (31.4%)	
Age (years)	16-25	10 (4.8%)	22 (10.6%)	32 (15.45%)	0.0422
	26-35	20 (9.6%)	21 (10.1%)	41 (19.8%)	
	36-45	13 (6.2%)	54 (26%)	67 (32.3%)	
	46-55	27 (13%)	40 (19.3%)	67 (32.3%)	
Patient category	Cases transferred to TB ward	12 (5.7%)	13 (6.2%)	25 (12%)	0.7454
	MDR-TB	30 (14.4%)	26 (12.5%)	56 (27%)	
	Pre-XDR-TB	24 (11.5%)	27 (13%)	51 (24%)	
	Fluoroquinolone resistant	24 (11.5%)	17 (8.2%)	41 (19.8%)	
	Fluoroquinolone sensitive	20 (9.6%)	14 (6.7%)	34 (16.4%)	

Prevalence of TB based on gender

**Figure 1:**
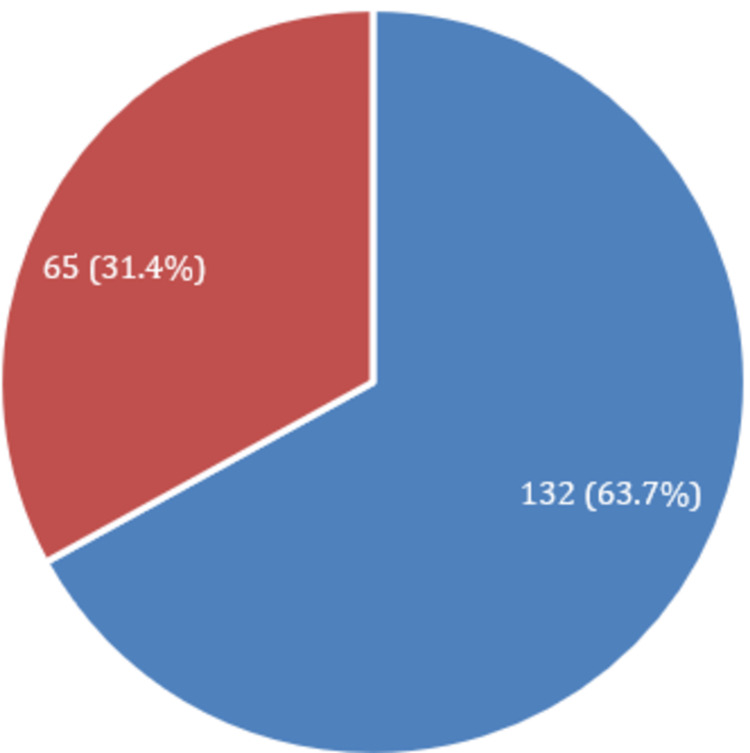
Prevalence of tuberculosis based on gender Blue color indicates male (n%): 132 (63.7%); Red color indicates female (n%): 65 (31.4%)

Out of 207 patients, 132 (63.76%) were male, and 65 (31.4%) were female. The high rate of infection in males might be due to smoking and tobacco use, which could increase the risk of TB infection. The graphical data of the study on a gender basis is provided in Figure 1.

Prevalence of TB based on age

**Figure 2:**
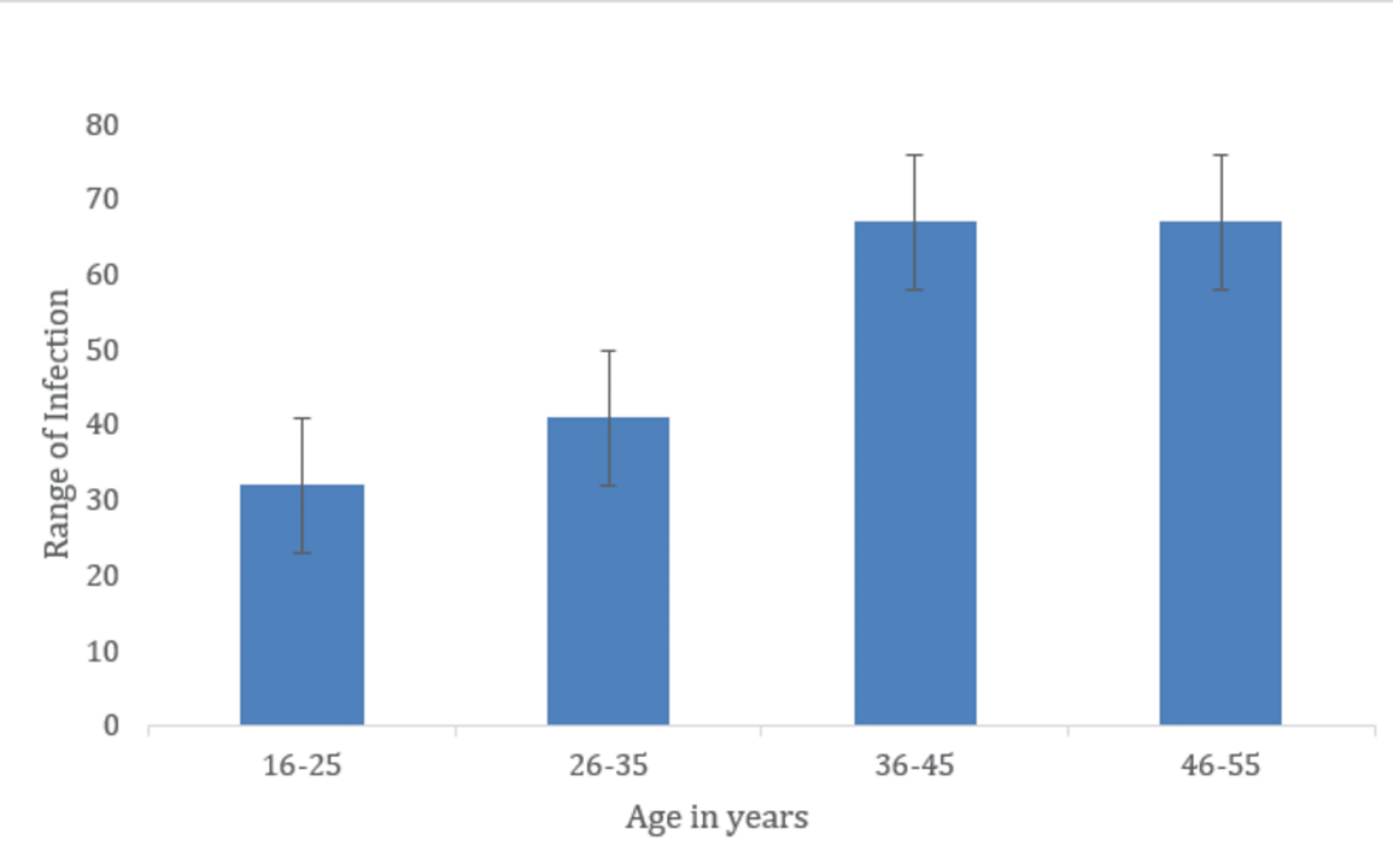
Prevalence of tuberculosis based on age The X-axis indicates the age in years and the Y-axis indicates the infection range Age (n%): 16-25, 32 (15.4%); 26-35, 41 (19.8%); 36-45, 67 (32.3%); 46-55, 67 (32.3%)

TB infection was categorized based on age, from 16 to 55. Notably, there is a sudden increase in patients aged 36-55 (Figure 2). This range of infections might be due to altered immune responses, defects in immune cells, or diabetic conditions.

Prevalence of TB based on drug resistance

**Figure 3:**
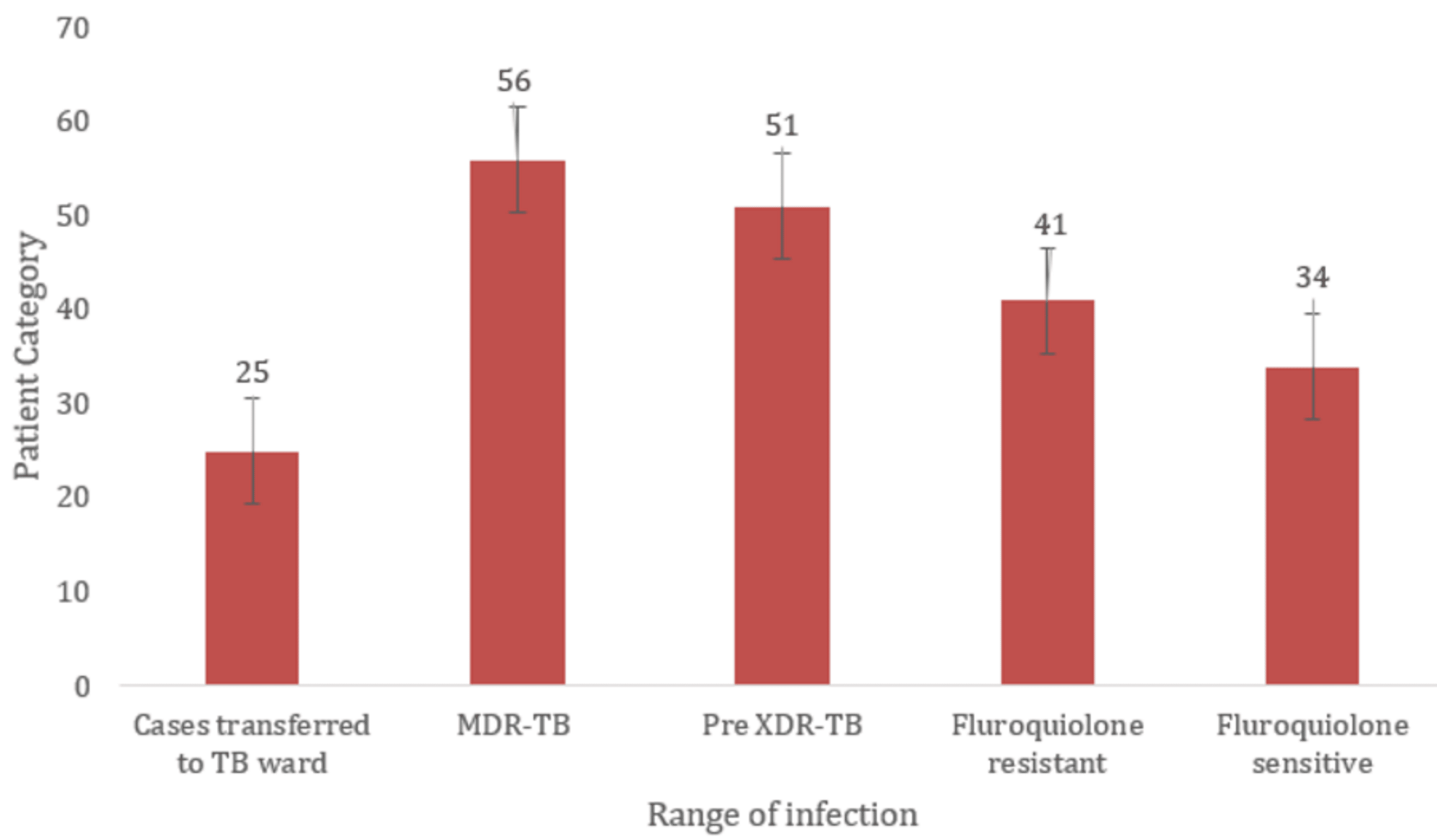
Prevalence of TB based on drug resistance The X-axis indicates the range of infection and the Y-axis indicates the patient category Cases transferred to the TB ward (n%): 25 (12%); MDR-TB (n%): 56 (27%); Pre-XDR-TB (n%): 51 (24%); Fluoroquinolone resistance (n%): 41 (19.8%); Fluoroquinolone-sensitive cases (n%): 34 (16.4%) TB: Tuberculosis; MDR-TB: Multidrug-resistant tuberculosis; XDR-TB: Extensively drug-resistant tuberculosis

In the total population of patients (n = 207) after phenotypic confirmation, 25 (12%) new cases were transferred to the TB ward. A high rate of TB infection was found in MDR-TB (56 cases, or 27%) and pre-XDR-TB (51 cases, or 24%). There was an increase in fluoroquinolone resistance (41 cases, or 19.8%), and some fluoroquinolone-sensitive cases (34 cases, or 16.4%) were also observed (Figure 3).

## Discussion

In the ongoing pursuit of novel therapeutic options for TB, historical research provides valuable insights. The discovery of promin, a dapsone derivative, in 1940 marked the first instance of potential efficacy demonstrated in an animal model (guinea pigs). Despite these promising initial findings, promin was never clinically evaluated in humans [16]. Linezolid is a potential antibiotic used to treat specific types of DR-TB. However, the optimal dosage of linezolid treatment remains questionable. The initial dosage of 600 mg appears to be well-tolerated, but the ideal treatment duration and the potential benefits of reducing the dosage later in treatment are still unclear [17]. Since there is a need to move beyond promising animal studies and ensure rigorous testing before relying on new drugs to combat TB, particularly in the face of increasingly resistant strains, traditional TB control methods like the Bacillus Calmette-Guérin (BCG) vaccine and chemoprophylaxis have become ineffective for resistant strains [18]. This highlights the crucial steps between initial discovery and effective treatment, especially for the ongoing challenges posed by MDR-TB. Opportunistically, research into newer oxazolidinones is ongoing; the next-generation antibiotic shows improved safety and tolerability profiles compared to linezolid [19].

The term "MDR-TB" notably indicates the increased susceptibility of tuberculin bacilli to isoniazid and rifampicin, the two most effective first-line TB medications [20]. The current standard treatment for MDR-TB might pose a significant risk for pre-XDR-TB patients who are resistant to fluoroquinolones and may not receive fully effective medications with the typical MDR-TB regimen. The two potential consequences of this condition are that the use of less effective drugs in pre-XDR-TB patients could further exacerbate existing drug resistance [21]. This is due to inappropriate treatment adherence, particularly missing doses of these crucial medications, which can contribute to the development of MDR-TB. Even more concerning is pre-XDR-TB, defined by resistance to a broader range of drugs, including fluoroquinolones [22]. This highlights the importance of rigorous pre-clinical and clinical trial phases to ensure the safety and efficacy of new anti-TB drugs before widespread human administration [23,24]. Fortunately, emerging treatment options for MDR/rifampicin-resistant TB (RR-TB) and pre-XDR-TB show promise for improved outcomes in combating these drug-resistant strains [25].

A recent update of December 2022 to the WHO treatment guidelines for DR-TB recommended a shorter six-month treatment regimen (BPaL/M or BPaL), incorporating the novel drug pretomanid alongside bedaquiline and linezolid, with or without the combination of moxifloxacin, compared to the previously standard nine-month or longer treatment regimens that could extend up to 18 months [11,26]. However, in several cases, like extensive lung disease or specific types of extrapulmonary TB, longer treatment durations might still be necessary [9]. This treatment regimen was supported by two trials: the TB-PRATECAL trial and the ZeNix trial, both of which showed a success rate of 80%-90%. The TB-PRATECAL trial demonstrated significantly improved treatment success rates with the BPaL/M regimen (86%-88%) at six months compared to current standard regimens (52%). Additionally, the trial reported lower rates of treatment failure, death, and patient loss of follow-up. The ZeNix trial confirmed the high success rates of BPaL (89%) observed in the Nix-TB trial, even when administered to pre-XDR-TB cases. This finding held true with a reduced linezolid dose (600 mg) and fewer reported adverse events [13,27]. From 2015 to 2018, first-line DST rose from 9% to 26%, and second-line drugs rose from 22% to 56% [28]. The study predicted a significant increase in the use of BPaLM regimens for treating drug-resistant patients, with projections indicating it will become the dominant treatment option by 2024 and reach 78% of patients by 2026 [29]. However, ensuring widespread access to new, more effective treatments requires substantial national efforts. Scaling up treatment programs, improving case-finding efforts, strengthening DST capabilities, and implementing these new regimens are crucial to ensuring all eligible patients benefit from these advancements and contribute to achieving global TB elimination goals. Additionally, actively engaging communities in capacity building and demand generation is essential for successful implementation and sustained progress in the fight against DR-TB.

Limitations

The retrospective analysis of TB treatment regimens possesses several limitations. The study findings are limited due to the exclusion of patients with significant comorbidities, including pregnancy, organ failure, uncontrolled HIV/AIDS, or active malignancy. Furthermore, the study only evaluated treatment regimens of six and nine months, while some cases necessitate extended regimens of up to 18 months. Additionally, the analysis focused solely on specific antibiotic groups and a limited age range. Future studies addressing these limitations would benefit from incorporating a more comprehensive patient population and evaluating the efficacy of long-term treatment regimens.

## Conclusions

The retrospective analysis of TB treatment regimens suggests the effectiveness of incorporating new drugs into a shorter six-month treatment regimen (BPaL/M or BPaL) with bedaquiline and linezolid. However, the retrospective study, within its limitations, needs further investigation. Future studies could involve prospective research to confirm these findings and explore treatment approaches for specific groups of patients, such as MDR-TB, pre-XDR-TB, and XDR-TB. These findings showed factors influencing treatment regimens and bacterial mechanisms involved in drug-resistance conditions, which aid in refining treatment strategies, contributing to more effective therapeutic practices, and the use of antibiotics.
